# Recurrent pleuropulmonary blastoma type III initially misclassified as embryonal rhabdomyosarcoma on limited biopsy: a case report with pathogenic DICER1 variant

**DOI:** 10.3389/fped.2026.1829736

**Published:** 2026-05-29

**Authors:** Suhaib Tawil, Nouraldeen Deeb, Wedad Alashwas, Hani Saleh, Khadra Salami, Alaa Jafar

**Affiliations:** 1 Faculty of Medicine, Al-Quds University, Jerusalem, Palestine; 2 Pediatric Department, Augusta Victoria Hospital, Jerusalem, Israel

**Keywords:** diagnostic pitfall, DICER1, IVADo, pediatric thoracic tumor, pleuropulmonary blastoma, pneumothorax

## Abstract

**Background:**

Pleuropulmonary blastoma (PPB) is a rare, aggressive pediatric thoracic malignancy and a sentinel tumor for DICER1 syndrome. Advanced PPB can mimic other pediatric sarcomas and complex cystic lung lesions, creating diagnostic and management pitfalls.

**Case presentation:**

A boy aged 2 years 11 months presented with progressive respiratory distress and fever due to a rapidly enlarging right hemithorax mass with marked mediastinal shift, near-complete right lung collapse, and recurrent pneumothorax. Initial CT-guided biopsy was interpreted as embryonal rhabdomyosarcoma (ERMS) on limited material, and systemic therapy was started with VAC, later escalated to VDC because of poor response and ongoing life-threatening mass effect. The clinical course was complicated by persistent/recurrent pneumothorax requiring multiple pleural interventions and eventual transfer for urgent surgical management. He underwent near-total resection followed by repeat resection for rapid local recurrence several weeks later. Definitive resection pathology established PPB, type III, and tumor next-generation sequencing detected a pathogenic DICER1 variant (germline status not yet confirmed). Postoperatively, chemotherapy was initiated with IVADo. A complete blood count obtained locally after return home showed severe cytopenias, prompting growth-factor support and close hematology-oncology follow-up.

**Conclusion:**

This case highlights that PPB should be prioritized in the differential diagnosis of aggressive pediatric intrathoracic masses with pneumothorax and mass effect, and demonstrates the value of adequate tissue sampling, expert pathology review, and early integration of molecular testing to prevent protocol misdirection and to trigger DICER1-directed genetic counseling and surveillance.

## Introduction

Pleuropulmonary blastoma (PPB) is a rare, aggressive pediatric malignancy arising from lung parenchyma and/or pleural surfaces, classically presenting in early childhood and existing along a developmental continuum from purely cystic (type I/regressed type Ir) to mixed cystic–solid (type II) and completely solid (type III) disease (1–4). Registry data emphasize both the age-related progression and the prognostic gradient across subtypes, with type III disease typically diagnosed around the preschool years and associated with higher risks of recurrence and metastasis compared with earlier cystic forms (2).

PPB is the sentinel tumor of the DICER1 tumor predisposition syndrome, an autosomal dominant condition with variable penetrance that confers risk for a spectrum of benign and malignant neoplasms (5–7). Accordingly, a diagnosis of PPB carries implications beyond the index tumor, including the need to consider germline testing, genetic counseling, and risk-adapted surveillance for the patient and at-risk relatives (6, 7).

Clinically, PPB can mimic infection, congenital lung malformations, and other pediatric thoracic malignancies; radiologic appearances are diverse and may include large multiloculated cystic components, hemorrhage, air–fluid levels, and secondary pneumothorax (3, 4, 8). Importantly, chest CT alone may be insufficient to reliably distinguish PPB from benign congenital cystic lesions, reinforcing the central role of definitive histopathology-often requiring adequate tissue sampling and expert review (8, 9). For advanced PPB (types II–III), contemporary management is multimodal, combining surgical resection with multiagent chemotherapy; collaborative data support doxorubicin-containing regimens and the IVADo protocol as commonly used approaches for type II/III disease (10–12).

Here, we present a preschool-aged child with a rapidly enlarging right hemithorax mass complicated by recurrent pneumothorax, initially interpreted on limited biopsy as embryonal rhabdomyosarcoma, but ultimately diagnosed as PPB type III with a pathogenic DICER1 variant. The case highlights diagnostic pitfalls, the value of multidisciplinary care and expert pathology review, and the educational importance of integrating tumor genetics into clinical decision-making and follow-up planning (13). This report is written in accordance with the CARE guideline framework for transparent case reporting (14).

## Case presentation

A boy aged 2 years 11 months was transferred from a local hospital for progressive respiratory distress and fever in the setting of a rapidly enlarging right thoracic mass. On arrival, he required low-flow oxygen (1 L/min by nasal cannula) and examination demonstrated markedly decreased right-sided air entry with tracheal deviation.

Initial laboratory testing showed leukocytosis (white blood cell count 12.5 × 10^9^/L), anemia (hemoglobin 7.1 g/dL; transfused), and normal platelet count (355 × 10^9^/L); electrolytes and renal function were normal.

**Figure 1:**
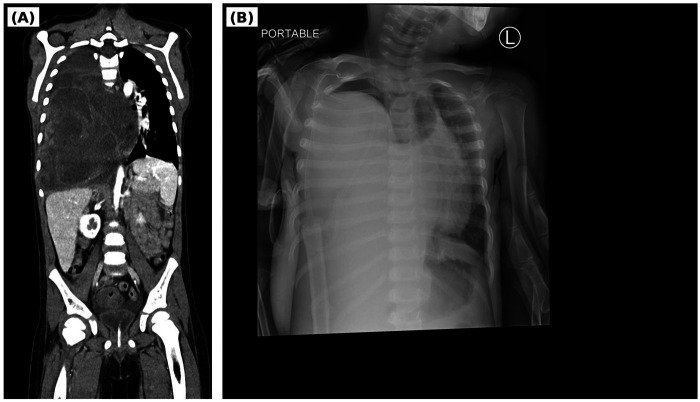
Representative imaging at presentation. **(A)** Coronal contrast-enhanced CT demonstrating a massive multiloculated predominantly cystic right hemithorax mass with marked mediastinal shift. **(B)** Portable chest radiograph demonstrating near-complete opacification of the right hemithorax with mass effect.

Contrast-enhanced chest CT performed at the referring hospital demonstrated a large heterogeneous mass occupying nearly the entire right hemithorax (approximately 12 × 11 × 10 cm). The lesion was predominantly cystic with internal septations and high-attenuation components suspicious for hemorrhage, with an internal gas locule and no gross calcification. There was complete collapse of the right lung with moderate right pneumothorax, occlusion of the right main bronchus, and marked mediastinal and cardiac shift to the left; a small pleural effusion was present. No lymphadenopathy, bone lesions, or contralateral pulmonary abnormalities were reported. Representative imaging findings are shown in Figure 1.

Repeat CT after transfer demonstrated interval enlargement to approximately 15 × 11 × 12 cm, described as a complex multiloculated cystic and soft-tissue mass arising from the pleura of the right lower lobe with enhancing soft-tissue components, thick septations, and an air-fluid level. No definite chest wall invasion was identified; right apical pneumothorax and a small pleural effusion persisted, with continued mass effect on mediastinal structures.

Although the definitive post-treatment resection specimen was classified as PPB type III, the predominantly cystic and septated appearance on initial imaging suggests that the pretreatment lesion may have fallen within the type II/III spectrum; this distinction does not change the therapeutic approach in this case but is relevant to interpreting the imaging-pathology discrepancy.

Given a large right-sided pneumohydropneumothorax with mediastinal shift, a right chest tube was placed early in the initial hospitalization with radiographic improvement. A CT-guided biopsy performed during the initial diagnostic evaluation was initially interpreted as embryonal rhabdomyosarcoma (ERMS) based on limited material; the report noted that an alternative primitive tumor with rhabdomyosarcomatous differentiation could not be fully excluded. At the time the tumor was presumed ERMS, initial staging was recorded as T1b Nx M0. Subsequent fluorescence *in situ* hybridization testing on the biopsy material did not demonstrate rearrangements involving ETV6, SS18, CIC, FOXO1, or EWSR1.

Systemic therapy was initiated according to a VAC regimen (vincristine, actinomycin D, cyclophosphamide) during the initial hospitalization. The hospital course was complicated by recurrent and persistent right pneumothorax attributed to near-complete hemithoracic tumor burden with bronchial obstruction and failure of the right lung to re-expand. The patient required multiple pleural interventions, including needle decompression and additional chest-tube placements over the following weeks. Attempts to clamp the chest tube resulted in recurrent pneumothorax and desaturation, necessitating continued drainage and suction.

Interval imaging on during early treatment demonstrated only slight tumor reduction (reported approximately 14 × 10 cm compared with 15.5 × 11.0 cm) with persistent necrosis and limited improvement in right lung aeration. A PET scan performed during staging showed a hypermetabolic infiltrative right hemithoracic mass with a few hypermetabolic mediastinal lymph nodes. Despite ongoing chemotherapy, the tumor demonstrated poor clinical response with progressive respiratory compromise.

Several weeks into treatment, the patient developed recurrent febrile episodes requiring broad-spectrum antibiotics (piperacillin-tazobactam). Given poor response to VAC, chemotherapy was escalated to a VDC regimen on. Later in the treatment course, he deteriorated with severe respiratory distress requiring admission to the pediatric intensive care unit (PICU) and high-flow nasal cannula (HFNC) support. He was transferred to a tertiary center for urgent surgical management.

The patient underwent near-total resection of the right pulmonary/pleural tumor after clinical deterioration and transfer for urgent surgical management; however, rapid local recurrence occurred, and repeat tumor resection was performed several weeks later. Postoperatively, he required PICU monitoring and transient HFNC support, later weaned to room air. Residual tumor burden remained in the subpulmonic region extending toward the diaphragm; a pleural drain was placed and subsequently removed.

Definitive histopathology from the resection specimen established the diagnosis of pleuropulmonary blastoma (PPB), type III. Immunohistochemistry demonstrated diffuse vimentin positivity with scattered desmin-positive large cells; calponin, myogenin, MyoD1, AE1/AE3, p40, and CD10 were negative, and Ki-67 was approximately 15%-20% in viable tumor areas. Treatment effect was present (approximately 40% residual viable tumor, 50% necrosis, and ∼10% fibrosis/inflammation), and the inked pulmonary resection margin was free of tumor. Tumor next-generation sequencing detected a pathogenic DICER1 missense variant, c.5425G>A (p.Gly1809Arg), with a variant allele frequency of 91.45%. A second DICER1 alteration, including a loss-of-function variant, was not reported in the available tumor NGS report. Germline status was not yet confirmed at the time of writing, prompting referral for genetic counseling and germline testing. Family history was negative for similar tumors, including pleuropulmonary blastoma. A summary of pathology and molecular findings is provided in Table 1.

**Table 1:** Pathology and molecular testing summary.

Specimen	Key histopathology	IHC	FISH/NGS
CT-guided biopsy	Reported as ERMS; limited/scant tissue with note that other primitive tumor with rhabdomyosarcomatous differentiation could not be excluded.	Reported focal positivity for desmin, myogenin, and MyoD1 (per original report).	FISH: negative for rearrangements involving ETV6, SS18, CIC, FOXO1, and EWSR1.
Pulmonary/pleural resection	Definitive diagnosis: pleuropulmonary blastoma (PPB), type III; treatment effect present (−40% viable tumor; 50% necrosis; −10% fibrosis/inflammation). Margin free.	Vimentin positive; scattered desmin-positive large cells. Calponin, myogenin, MyoD1, AE1/AE3, p40, and CD10 negative. Ki-67 −15–20% in viable areas.	Tumor NGS: DICER1 c.5425G> A (p.Gly1809Arg), pathogenic missense variant, VAF 91.45%, detected. A second DICER1 alteration, including a loss-of-function variant, was not reported in the available tumor NGS report. Germline status was not yet confirmed.

Following multidisciplinary discussion, chemotherapy was initiated according to an IVADo regimen, with subsequent cycles planned per protocol. The postoperative course included intermittent fevers treated with antibiotics with subsequent de-escalation; prophylactic trimethoprim-sulfamethoxazole was continued. He received packed red blood cell transfusion for postoperative hemoglobin of 5.9 g/dL with subsequent improvement. A complete blood count obtained locally shortly after return home demonstrated marked cytopenias (hemoglobin 7.12 g/dL, white blood cell count 0.88 × 10^9^/L with absolute neutrophil count −0.10 × 10^9^/L, and platelets 7.1 × 10^9^/L), and he was prescribed filgrastim support and close outpatient follow-up. At the last available follow-up, he was clinically stable on room air and continuing protocol-directed therapy with planned hematology-oncology review. A chronological summary of the clinical course is provided in Table 2.

**Table 2:** Chronological clinical timeline.

Time since evaluation	Key events	Imaging/key findings	Management
Initial evaluation	CT-guided biopsy of right thoracic mass	Large right hemithorax mass	Biopsy interpreted as ERMS on limited material
1 day later	Respiratory distress with pneumohydropneumothorax	Mediastinal shift; right lung collapse	Right chest tube placed
3 day later	Systemic therapy initiated	–	VAC chemotherapy started
Two weeks	Metastatic evaluation	PET: hypermetabolic right mass; few mediastinal nodes	Continued VAC
Three weeks	Recurrent pneumothorax	Persistent pneumothorax	Needle decompression; second chest tube
Three weeks	Interval CT response assessment	Tumor −14 × 10 cm with necrosis (slight reduction)	Supportive care; ongoing chemotherapy
Four weeks	Febrile episode	–	Piperacillin-tazobactam restarted; surgery deferred
Five weeks	Large pneumothorax with non-functioning tube	CXR: large pneumothorax	Chest tube replaced (third tube)
Six weeks	Poor response to VAC	Progressive tumor burden	Escalated to VDC regimen
Six weeks	Clinical deterioration	Progressive right hemithorax occupation	ICU admission; high-flow nasal cannula (HFNC); transfer for surgery
Seven weeks	Near-total tumor resection	Residual subpulmonic disease	Surgical resection
Eight weeks	Rapid local recurrence	–	Repeat tumor resection; pediatric ICU (PICU) care
Eight weeks and a half	Pre-chemotherapy imaging	CT: progression with compression of right main bronchus/mediastinal structures	Initiated IVADo chemotherapy
Nine weeks	Postoperative course	–	Weaned to room air; antibiotics de-escalated; prophylaxis continued
Nine weeks	Hematologic toxicity/cytopenias	CBC: Hb 7.12 g/dL; WBC 0.88 × 10^9^/L; ANC −0.10 × 10^9^/L; Plt 7.1 × 10^9^/L	Filgrastim and close follow-up

### Differential diagnosis

The initial differential diagnosis for a rapidly enlarging mixed cystic–solid intrathoracic mass in a child of this age included congenital pulmonary airway malformation (CPAM), pleuropulmonary blastoma (PPB), and primary thoracic sarcomas (including rhabdomyosarcoma), with complicated infection (e.g., necrotizing pneumonia with pneumatoceles) considered less likely. The rapid interval growth, progressive mediastinal shift, near-total ipsilateral lung collapse, and recurrent pneumothorax were atypical for benign congenital lesions. Although the limited CT-guided biopsy initially supported embryonal rhabdomyosarcoma, known histopathologic overlap in small samples necessitated definitive surgical pathology and molecular correlation for accurate classification.

## Discussion

This case illustrates several high-yield clinical and educational lessons: (i) PPB should remain prominent in the differential diagnosis for a young child with a rapidly enlarging thoracic mass-particularly when complicated by pneumothorax and near-complete lung collapse; (ii) limited biopsy material can misclassify PPB as another small round/primitive sarcoma, including rhabdomyosarcoma, because rhabdomyoblastic differentiation is part of the PPB histologic spectrum; and (iii) the definitive diagnosis carries immediate implications for therapy selection and for genetic evaluation/surveillance due to frequent association with DICER1 (2–8).

### Diagnostic reasoning and the limits of imaging

PPB is uncommon, but it is among the most important malignant diagnoses to exclude in early childhood thoracic masses because delayed recognition can allow progression along the cystic-to-solid continuum and may worsen outcomes (2–4). Imaging can strongly suggest malignancy (e.g., massive size, mediastinal shift, hemorrhagic components, complex septations, or solid enhancing elements), yet PPB remains a radiologic “mimicker.” (8) In cystic presentations, the overlap with congenital pulmonary airway malformations is well recognized; even with modern CT interpretation, interrater agreement is imperfect and discriminating features may be unreliable (9). Although our patient ultimately had advanced disease, the initial presence of a predominantly cystic, septated lesion with hemorrhagic components and recurrent pneumothorax fits within the broad reported spectrum of PPB imaging appearances and underscores that “benign-appearing cystic” morphology does not exclude malignancy (4, 8, 9).

Educationally, this supports a practical approach for clinicians and radiologists: when prenatal imaging does not definitively establish a congenital diagnosis, when postnatal clinical behavior is aggressive (rapid enlargement, recurrent pneumothorax, progressive mediastinal shift), or when the lesion has suspicious internal architecture (solid nodules, thick septa, hemorrhage), early surgical evaluation and a pathway to definitive tissue diagnosis should be prioritized over prolonged observation (8, 9).

### Pathology pitfalls and the value of expert review

A major teaching point in this case is the initial interpretation of limited biopsy as embryonal rhabdomyosarcoma. This scenario is credible and well described: PPB type II/III can contain areas that resemble embryonal rhabdomyosarcoma or other high-grade sarcomatous elements, and small biopsies may sample only a non-representative component (4, 8). The risk is not merely academic-misclassification can lead to selection of a disease-specific protocol that may not optimally address PPB biology and local control needs. Registry experience demonstrates that central pathology review can reclassify a meaningful fraction of suspected cases as alternative entities; in the large International PPB Registry series, approximately one-fifth of cases submitted for review were ultimately “another entity,” reinforcing why second-opinion review is not optional in atypical pediatric thoracic tumors (2).

From an educational standpoint, two safeguards deserve emphasis. First, when clinicoradiologic behavior is discordant with the expected course (e.g., minimal response to protocol-directed therapy, persistent life-threatening mass effect), teams should trigger an explicit “diagnostic time-out” that includes re-review of histology, consideration of repeat sampling, and/or referral for expert pathology consultation (4, 8). Second, molecular testing should be integrated early in the diagnostic workflow when a primitive intrathoracic tumor arises in the age range and morphologic context compatible with PPB, because it can support reclassification and guide downstream genetic counseling (5–7).

### Therapeutic implications and the role of local control

For type II/III PPB, multimodality therapy is the standard conceptual approach: aggressive local control (often surgery, sometimes staged) combined with systemic multiagent chemotherapy (4, 10, 11). Outcomes and prognostic factors reported by collaborative groups support the importance of complete resection when feasible and the benefit of doxorubicin-containing chemotherapy regimens (10). More recent International PPB/DICER1 Registry data indicate that the IVADo regimen (ifosfamide, vincristine, actinomycin-D, doxorubicin) has been associated with similar-to-improved survival compared with historical controls for type II/III disease, and now serves as a widely referenced benchmark regimen (12). The EXPeRT/PARTNER recommendations likewise emphasize multidisciplinary planning, adequate diagnostic workup (including brain imaging for advanced disease), and integration of surgery with systemic therapy for type II/III PPB (11).

In this patient, near-total resection followed by rapid recurrence and a second resection highlights the biologic aggressiveness of advanced PPB and the clinical reality that complete upfront resection is not always feasible when tumor burden is massive and physiology is tenuous. Contemporary guidance supports individualized sequencing (neoadjuvant vs. upfront surgery) based on anatomy, operability, and clinical stability, underscoring the need for early tertiary-center involvement and repeated tumor board reassessment as the disease evolves (4, 11, 12). The subsequent transition to an IVADo-based approach is aligned with these evidence-informed recommendations for type III PPB (11, 12).

### DICER1 implications: genetics, counseling, and surveillance

The finding of a pathogenic DICER1 variant in tumor sequencing should prompt a structured genetics pathway. In this patient, the pathogenic DICER1 variant was detected on tumor sequencing; germline testing had not yet been completed at the time of writing. DICER1 syndrome is a recognized tumor predisposition condition, and germline testing (with appropriate pre-test counseling) is needed to determine whether the patient is a constitutional carrier and whether relatives may be at risk (5–7). If sequencing-based germline testing is negative, further genetics review should consider whether deletion/duplication analysis or other methods are needed to evaluate for germline DICER1 alterations that may be difficult to detect by standard sequencing alone. Surveillance recommendations for confirmed carriers vary by guideline and require balancing earlier detection against the burdens of radiation exposure and repeated imaging; consensus-based frameworks propose periodic clinical assessment and organ-targeted imaging in early childhood, with consideration of low-dose chest CT at selected ages in some protocols (13). Beyond thoracic disease, families should be educated about the broader DICER1 tumor spectrum and the rationale for age-appropriate screening strategies (6, 7, 13).

### CARE checklist alignment and educational takeaways

This case was structured to align with CARE principles-clear diagnostic assessment, a chronological timeline, and follow-up status-to support transparent learning and clinical transferability (14). The educational value is strongest at the “interfaces” of care: radiology–surgery (when pneumothorax and mass effect drive urgent decisions), pathology–oncology (when small biopsies risk misclassification), and oncology–genetics (when tumor sequencing informs family risk). Clinicians encountering similar presentations can take away a simple rule: in a young child with an aggressive, complex cystic/solid intrathoracic mass-especially with recurrent pneumothorax-PPB must be actively considered, tissue adequacy and expert pathology review are essential, and DICER1-directed counseling/surveillance planning should be initiated once PPB enters the diagnostic frame (2–4, 8, 11–13).

Clinical lessons. In aggressive pediatric intrathoracic tumors, discordance between radiologic behavior and treatment response should trigger an early multidisciplinary “diagnostic time-out,” including reassessment of tissue adequacy and consideration of expert pathology review and molecular testing. In PPB, rhabdomyoblastic differentiation may lead to misclassification as embryonal rhabdomyosarcoma on limited biopsy, and accurate diagnosis directly impacts multimodal therapy planning and prompts DICER1-directed genetic counseling and germline evaluation.

## Conclusion

This case of a rapidly progressive right hemithorax mass in a preschool-aged child underscores that pleuropulmonary blastoma (PPB) should be actively considered when a young patient presents with a large complex intrathoracic lesion complicated by pneumothorax and profound mass effect. Misclassification on limited biopsy as embryonal rhabdomyosarcoma can occur because PPB may show rhabdomyoblastic differentiation; therefore, adequate tissue sampling, expert pathology review, and integration of molecular findings are critical to avoid protocol misdirection and to enable timely PPB-directed multimodality therapy (2–4, 8, 11, 12). Identification of a pathogenic DICER1 variant on tumor sequencing adds essential educational value, as it should trigger genetic counseling, consideration of germline testing, and risk-adapted surveillance for the patient and potentially affected relatives (5–7, 13). Overall, the report highlights a practical diagnostic “checkpoint” for clinicians: in aggressive pediatric thoracic tumors with discordant clinicoradiologic–pathologic features or suboptimal treatment response, an early multidisciplinary reassessment-including molecular testing-can be decisive for both oncologic outcomes and family-centered care.

## Patient perspective

The patient's family reported that the prolonged course with recurrent pneumothorax and escalating treatment was distressing, and they valued having a clear diagnosis and a defined plan including genetic counseling and follow-up.

## Data Availability

The original contributions presented in the study are included in the article/Supplementary Material, further inquiries can be directed to the corresponding author/s.
